# Analysis of drug-susceptibility patterns and gene sequences associated with clarithromycin and amikacin resistance in serial *Mycobacterium abscessus* isolates from clinical specimens from Northeast Thailand

**DOI:** 10.1371/journal.pone.0208053

**Published:** 2018-11-29

**Authors:** Pimjai Ananta, Irin Kham-ngam, Ploenchan Chetchotisakd, Prajuab Chaimanee, Wipa Reechaipichitkul, Wises Namwat, Viraphong Lulitanond, Kiatichai Faksri

**Affiliations:** 1 Department of Microbiology, Faculty of Medicine, Khon Kaen University, Khon Kaen, Thailand; 2 Clinical Laboratory Unit, Srinagarind Hospital, Faculty of Medicine, Khon Kaen University, Khon Kaen, Thailand; 3 Research and Diagnostic Center for Emerging Infectious Diseases, Khon Kaen University, Khon Kaen, Thailand; 4 Department of Medicine, Faculty of Medicine, Khon Kaen University, Khon Kaen, Thailand; St Petersburg Pasteur Institute, RUSSIAN FEDERATION

## Abstract

*Mycobacterium abscessus* is an important infectious agent highly associated with drug resistance and treatment failure. We investigated the drug resistance situation of *M*. *abscessus* in Northeast Thailand and the possible genetic basis for this. Sixty-eight *M*. *abscessus* clinical isolates were obtained from 26 patients at Srinagarind Hospital during 2012–2016. Drug susceptibility tests and sequencing of *erm(41)*, *rrl* and *rrs* genes were performed. *Mycobacterium abscessus* was resistant to 11/15 antibiotics (nearly 100% resistance in each case). Partial susceptibility to four antibiotics was found (amikacin, tigecycline, clarithromycin and linezolid). Non-*massiliense* subspecies were significantly associated with clarithromycin resistance (p<0.0001) whereas *massiliense* subspecies were associated with tigecycline resistance (p = 0.028). Inducible clarithromycin resistance was seen in 22/68 (32.35%) isolates: 21 of these isolates (95.45%) belonged to non-*massiliense* subspecies and resistance was explicable by the T28C mutation in *erm(41*). Inducible clarithromycin resistance was found in one isolate of the *massiliense* subspecies. Acquired clarithromycin resistance explicable by the A2271G/C mutation of *rrl* was seen in only 7/16 (43.75%) of strains. Inducible and acquired resistance mechanisms can be interchangeable during the course of infection. *Rrs* mutations were not associated with amikacin resistance in our study. Antibiotic resistance in subspecies of *M*. *abscessus* was reported from Northeast Thailand. Known resistance-associated mutations cannot explain all of the resistance patterns observed.

## Introduction

Nontuberculous mycobacteria (NTM) do not cause tuberculosis (TB) but can nevertheless cause life-threatening diseases. Although NTM infection exhibits similar characteristics to TB in terms of symptoms and AFB staining, different antibiotics are required for treatment. Some species of NTM have been found to be resistant to many types of antibiotics [1].

*Mycobacterium abscessus* is a rapidly growing mycobacterium that causes a wide spectrum of diseases in humans, including pulmonary, skin and soft tissue, and disseminated disease [2]. In addition, it is uniformly resistant to the standard anti-TB agents and is highly associated with treatment failure [3]. *Mycobacterium abscessus* has been classified into three subspecies based on whole-genome sequencing analysis: *M*. *abscessus* subsp. *abscessus*, *M*. *abscessus* subsp. *bolletii*, and *M*. *abscessus* subsp. *massiliense* [4].

The antibiotic regimen recommended for treatment of *M*. *abscessus* infection (ATS/IDSA guidelines 2007) is a multidrug macrolide-based therapy and hence clarithromycin is regarded as a key antibiotic [2]. In addition, natural susceptibility of *M*. *abscessus* to amikacin has been reported and hence inclusion of this antibiotic in the treatment regimen has been suggested [5]. Two main classes of clarithromycin resistance are acquired and inducible resistance [6, 7]. Acquired clarithromycin resistance is associated with point mutations at positions 2,058 and 2,059 of the *rrl* gene encoding the 23S rRNA [6]. Inducible resistance is conferred by the *erm(41)* gene, coding for erythromycin ribosomal methylases [7, 8]. Several studies have investigated the roles of *erm(41)* and *rrl* of *M*. *abscessus* in conferring resistance to clarithromycin [9–12], but with inconsistent results [13]. These mutations did not explain all clarithromycin-resistant *M*. *abscessus* strains [13] and other mechanisms might exist. Amikacin resistance is also partially explained by point mutations at positions 1,406 to 1,409 in the *rrs* gene encoding 16S rRNA [8, 14, 15].

Studies in a number of countries have reported the drug resistance situation for *M*. *abscessus* [16–18]. The few reports of the drug-resistance situation in *M*. *abscessus* infection in Thailand date to before 2005 [19–21]: further study to supplement and update these is needed.

Serially isolated strains of a bacterial pathogen provide a good model to investigate the acquired drug resistance mechanism and to monitor changes in drug susceptibility during the course of an NTM infection. So far, there has only been only one report (from Spain) investigating clarithromycin resistance in four serially isolated strains of *M*. *abscessus* [22].

We aim to update information on the drug resistance situation and to investigate mutations of genes associated with clarithromycin and amikacin resistance in serial isolates of *M*. *abscessus* from patients in Northeast Thailand.

## Material and methods

### Studied population and setting

Sixty-eight *M*. *abscessus* isolates were obtained from 26 patients at Srinagarind Hospital between 2012 to 2016. This hospital is a tertiary University Hospital in Khon Kaen Province and is the largest hospital in Northeast Thailand, serving patients from several provinces there. Relevant clinical, laboratory and demographic data were retrieved from medical record databases. This study was approved by the Khon Kaen University Ethics Committee for Human Research (HE591454). All specimens were fully anonymized before they were accessed. This study used left-over specimens without the information that could lead to identification of any study participant and no informed consent is required.

### Case definition

Among the 26 patients, 20 were regarded as suffering true NTM infection based on the criteria in the ATS/IDSA guidelines 2007 [2]; isolation from a sterile site (n = 10), had radiological data, isolation from sputum ≥2 times, with clinical symptoms and also supported by history of NTM treatment (n = 10). The remaining six patients were previously treated TB patients who did not match the criteria of true infection were therefore defined as possible colonization cases.

### Drug susceptibility testing

Drug susceptibility testing was performed using a RAPMYCOI Sensititre 96-well plate (Sensititre, Trek Diagnostic Systems, United Kingdom). The plates were incubated at 30°C for 5 days (except for the clarithromycin susceptibility test). Clarithromycin susceptibility was read at 3, 5 and 14 days. A reading at day 3 was used to test for inducible resistance according to the protocol previously described [7]. Inducible resistance was inferred by changes in MIC values from susceptible at day 3 to resistant at day 14. Strains with resistance status since day 3 were regarded as demonstrating acquired resistance. Interpretation of the results followed the Clinical and Laboratory Standards Institute (CLSI M24-A2) guidelines. *Staphylococcus aureus* ATCC29213 and *Mycobacterium abscessus* ATCC19977 were used as control strains.

### DNA extraction

All *M*. *abscessus* isolates were subcultured onto Löwenstein-Jensen media and incubated at 37°C for 7 days. Genomic DNA from *M*. *abscessus* isolates was extracted from multiple loopfuls of *M*. *abscessus* colonies using the cetyl-trimethyl-ammonium bromide-sodium chloride (CTAB) method [23].

### PCR and gene sequencing

We selected 3 genes (*rrs*, *erm(41*) and *rrl*) to investigate the resistance-related genetics of two antibiotics, amikacin and clarithromycin (inducible and acquired resistance in the latter). Quick Taq HS DyeMix (Taq-based 2x master mix PCR reagent containing all components for PCR except primers and template DNA) were used in PCR for sequencing. The PCR conditions for amplification of all genes were as follows; pre-denaturation at 95°C for 5min; 35 cycles of 95°C for 1 min, 63°C (64°C for *erm(41)* and for *rrl*) for 1 min, 72°C for 1.5 min and final elongation at 72°C for 10 min. **Table 1** lists the primer sequences that were newly designed in our study. The PCR products were sent for sequencing using Sanger sequencing (BioBasic Inc., Canada) with an Automated Sequencer ABI Prism 3730XL.

**Table 1:** Primers used for gene sequencing of *erm(41)*, *rrl* and *rrs*.

Genes	Primers	Sequences (5’ to 3’)	Product length (bp)
*erm(41)*	ERM_F	TGCCCCGATATCTTTGGAGC	620
	ERM_R	GATTCCACCGGTTAGGCCG	
*rrl*	RRL_F	AACTTCGGGAGAAGGGGGA	1100
	RRL_R	AGAAACCTGGTCTTGGAATAGG	
*rrs*	RRS_F	GACAAACAATTCTTTTGACAGTTG	1600
	RRS_R	ATGTTCCCTAGTTCATTCGAC	

### Data analysis

Subspecies of *M*. *abscessus* were identified by their *erm(41)* gene sequences. A 2-bp deletion of nucleotides 64–65 and a 274-bp deletion of nucleotides 159–432 in this gene (based on genomic positions in the *M*. *abscessus* reference strain ATCC19977, GenBank accession number CU458896) distinguished *M*. *abscessus* subspecies *massiliense* from the other two subspecies [7, 24, 25]. Comparisons of drug susceptibility patterns between *massiliense* and non-*massiliense* subspecies were calculated as percentage and proportion. Drug susceptibility patterns and sequences of associated genes were analyzed in serially isolated strains exhibiting changing drug susceptibility (within-patient analysis) and between drug-susceptible vs drug-resistant groups of isolates (between group analysis). A correlation analysis of SNPs and MIC level of antibiotics was performed. A chi-square test or Fisher’s exact test were used for the comparison of categorical variables. P<0.05 was considered significant. Demographic and epidemiological data, as well as the clinical information, were analyzed. All statistical analyses were performed using SPSS version 16.0.

## Results

### Drugs resistance situation of *M*. *abscesses* in Northeast Thailand

The drug susceptibility pattern of *M*. *abscessus* isolates from clinical specimens of 26 cases (n = 68) is shown in **Table 1**. Our *M*. *abscessus* isolates exhibited nearly 100% resistance to 11/15 antibiotics. Partial susceptibility was noted to only 4 antibiotics (82.35% of isolates were susceptible to amikacin, 50% to tigecycline, 48.53% to clarithromycin and 14.71% to linezolid) (**Table 2**).

**Table 2:** Drug susceptibility patterns of *Mycobacterium abscessus* isolates from clinical specimens of patients in Northeast Thailand.

Drug susceptibility patterns (n/%) (Total = 68 isolates)				Interpretation criteria (μg/ml)		
Drugs in the guidelines ^a^	S	I	R	S	I	R
Amikacin	**56 (82.35%)**	7 (10.29%)	5 (7.35%)	≤16	32	≥64
Cefoxitin	0 (0%)	11 (16.18%)	57 (83.82%)	≤16	32–64	≥128
Ciprofloxacin	0 (0%)	0 (0%)	68 (100%)	≤1	2	≥4
Clarithromycin	**33 (48.53%)**	6 (8.82%)	29 (42.64%)	≤2	4	≥8
Doxycycline	1(1.47%)	5 (7.35%)	62 (91.18%)	≤1	2–4	≥8
Imipenem ^b^	0 (0%)	0 (0%)	68 (100%)	≤4	8–16	≥32
Linezolid	**10 (14.71%)**	16 (23.53%)	42 (61.76%)	≤8	16	≥32
Moxifloxacin	0 (0%)	2(2.94%)	66 (97.05%)	≤1	2	≥4
Tobramycin	0 (0%)	1(1.47%)	67 (98.53%)	≤2	4	≥8
SXT	0 (0%)	0 (0%)	68 (100%)	≤2/38	-	≥4/76
Drugs out of guidelines ^c^	Low	Moderate	High	Low	Moderate	High
AMC	0 (0%)	0 (0%)	68 (100%)	≤16	32	≥64
Cefepime	0 (0%)	0 (0%)	68 (100%)	≤8	16	≥32
Ceftriaxone	0 (0%)	0 (0%)	68 (100%)	≤16	32	≥64
Minocycline	0 (0%)	0 (0%)	68 (100%)	≤2	4	≥8
Tigecycline	**34 (50%)**	15 (22.06%)	19 (27.94%)	≤1	2	≥4

^**a**^ CLSI M24-A2 guidelines

^b^ Natural resistance

^**c**^ Antibiotics not described in the M24-A2 guidelines and levels of resistance interpreted here as low, moderate and high. S = Susceptible, I = Intermediate, R = Resistant, Low = Low resistance, Moderate = Moderate resistance, High = High resistance, SXT = Trimethoprim/sulfamethoxazole, AMC = Amoxicillin/clavulanic acid

### Association between *massiliense* and non-*massiliense* subspecies of *M*. *abscessus* and drug-susceptibility patterns

We found that non-*massiliense* subspecies were significantly associated with clarithromycin resistance (p<0.0001) whereas the *massiliense* subspecies was significantly associated with tigecycline resistance (high resistance combined with intermediate resistance) (**Table 3**). However, both subspecies categories were highly susceptible to amikacin and moderately resistant to linezolid.

**Table 3:** Comparisons of drug susceptibility pattern in *massiliense* and non-*massiliense* subspecies of *M*. *abscessus* isolated from clinical specimens.

Drug susceptibility patterns of *M*. *abscessus* subspecies; n (%)							P-values among S, R and I	S vs combined I and R
Antibiotics in the guidelines	*massiliense* subspecies (n = 37)			non-*massiliense* subspecies (n = 31)				
	S	I	R	S	I	R		
Amikacin	**33 (89.19%)**	2 (5.41%)	2 (5.41%)	**23 (74.19%)**	5 (16.13%)	3 (9.68%)	0.251	0.106
Cefoxitin	0 (0%)	4 (10.81%)	33 (89.19%)	0 (0%)	7 (22.58%)	24 (77.42%)	0.189	NA
Ciprofloxacin	0 (0%)	0 (0%)	37 (100%)	0 (0%)	0 (0%)	31 (100%)	NA	NA
Clarithromycin	**26 (70.27%)**	2 (5.41%)	9 (24.32%)	7 (22.58%)	4 (12.90%)	**20 (64.52%)**	**<0.0001**	**<0.0001**
Doxycycline	0 (0%)	2 (5.41%)	35 (94.59%)	1 (3.23%)	3 (9.68%)	27 (87.10%)	0.424	0.456
Imipenem	0 (0%)	0 (0%)	37 (100%)	0 (0%)	0 (0%)	31 (100%)	NA	NA
Linezolid	3 (8.11%)	9 (24.32%)	**25 (67.57%)**	7 (22.58%)	7 (22.58%)	17 (54.84%)	0.239	0.167
Moxifloxacin	0 (0%)	0 (0%)	37 (100%)	0 (0%)	2 (6.45%)	29 (93.55%)	NA	NA
SXT	0 (0%)	0 (0%)	37 (100%)	0 (0%)	0 (0%)	31 (100%)	NA	NA
Tobramycin	0 (0%)	0 (0%)	37 (100%)	0 (0%)	1 (3.23%)	30 (96.77%)	NA	NA
Antibiotics out of the guidelines	Low	Moderate	High	Low	Moderate	High	Among 3 patterns	S vs combined I and R
AMC	0 (0%)	0 (0%)	37 (100%)	0 (0%)	0 (0%)	31 (100%)	NA	NA
Cefepime	0 (0%)	0 (0%)	37 (100%)	0 (0%)	0 (0%)	31 (100%)	NA	NA
Ceftriaxone	0 (0%)	0 (0%)	37 (100%)	0 (0%)	0 (0%)	31 (100%)	NA	NA
Minocycline	0 (0%)	0 (0%)	37 (100%)	0 (0%)	0 (0%)	31 (100%)	NA	NA
Tigecycline	14 (37.84%)	**9 (24.32%)**	**14 (37.84%)**	20 (64.52%)	6 (19.35%)	5 (16.13%)	0.066	**0.028**

SXT = Trimethoprim/sulfamethoxazole, AMC = Amoxicillin/clavulanic acid * the *massiliense* subspecies was defined by characteristic deletions in the *erm(41)* gene (a 2-bp deletion of nucleotides 64–65 and a 274-bp deletion of nucleotides 159–432). Sequence data for *erm(41)* were available for 64/68 isolates, enabling classification to subspecies level. The subspecific predictions for the remaining 4 isolates were based on MLST data (Kham-ngam I. et al, unpublished). The interpretation of drug susceptibility patterns is described in **Table 1**.

### Changes in DNA sequences and MIC levels in isolates serially sampled from individual patients

**Fig 1:**
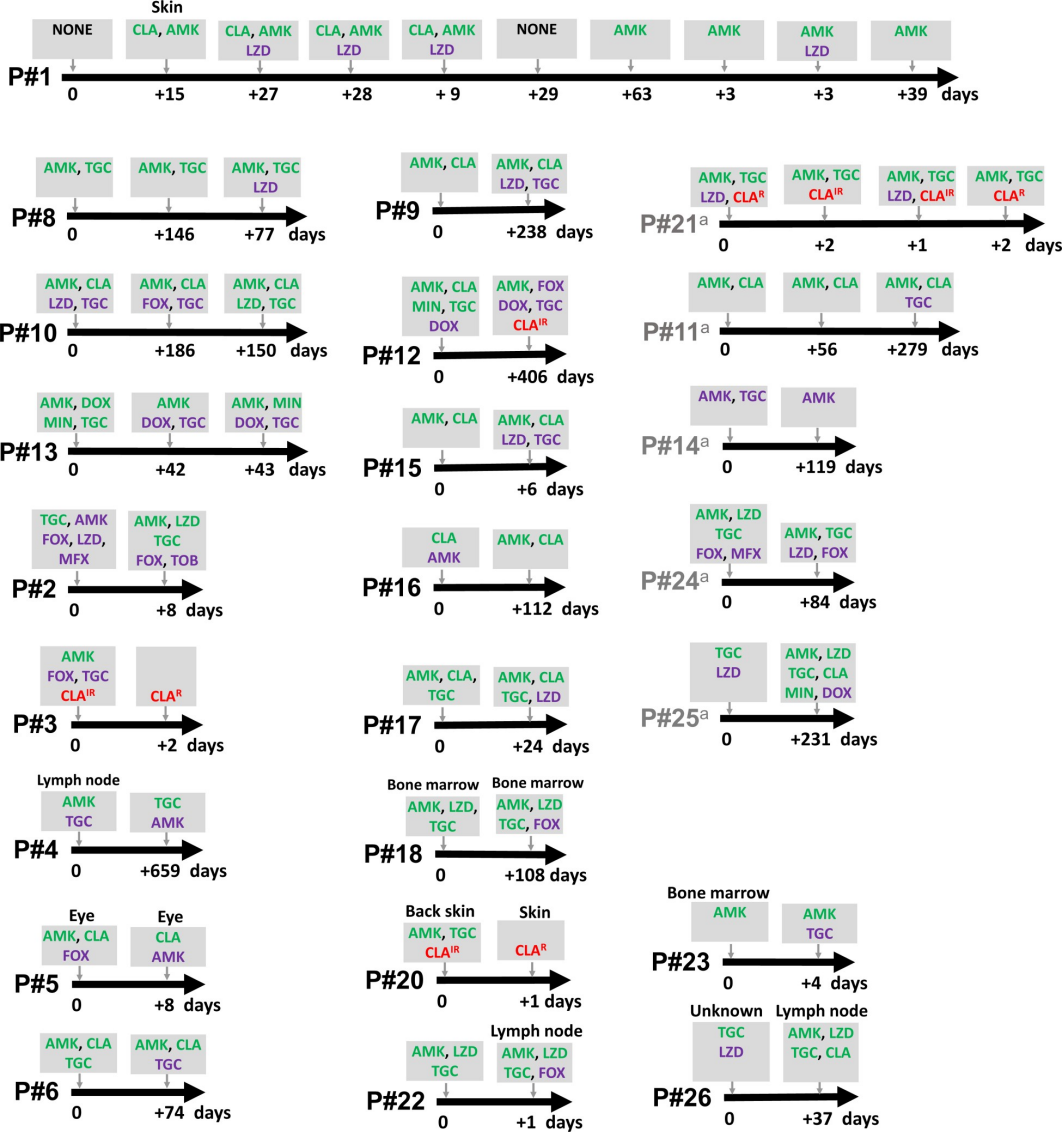
Drug susceptibility patterns of *M*. *abscessus* serially isolated from individual patients (P#1-P#26). Only susceptible (green letters) or intermediate (purple letters) levels of drug sensitivity are shown, except for “CLA” for which “IR” (inducible resistance) and “R” (acquired resistance) are shown. Organ sites are shown except pulmonary sites. Reinfection or recolonization strains (different strain among serial isolates– 2 patients) are excluded. “NONE” refers to resistance to all 15 antibiotics. The timeline shows number of days between successive samplings. ^a^ These cases were defined as colonization. AMK = Amikacin, FOX = Cefoxitin, CLA = Clarithromycin, DOX = Doxycycline, LZD = Linezolid, MFX = Moxifloxacin, Min = Minocycline, TGC = Tigecycline.

A change in MIC levels for all 15 antibiotics was often detected between serial isolates from individuals, regardless of whether that individual was diagnosed as infected or colonized. MIC levels for 7/15 antibiotics were found to increase or to decrease over different sampling intervals in serially isolated strains (**S1 Table**). Drug susceptibility of serial isolates from each patient could also change from resistant to susceptible or susceptible to resistant (**Fig 1** and **S1 Table**). Inducible and acquired resistance to clarithromycin were interchangeable (**Fig 1**).

For two antibiotics, associated genes were sequenced (*erm(41)* and *rrl* for clarithromycin and *rrs* for amikacin). No particular genetic variant was associated with increased or decreased MIC levels (**Table 4**) and susceptibility patterns (resistance or susceptibility) (data not shown) in amikacin and clarithromycin.

**Table 4:** Association between gene sequence and MIC levels for clarithromycin and amikacin.

Antibiotics	Genes	Increased MIC		Decreased MIC	
		No. of isolates	Variants (No. of isolates)	No. of isolates	Variants (No. of cases)
Clarithromycin	*erm(41)*	11	De11A (10) ^a^, C54T (n = 1), T55A (n = 1), De26A (n = 1)	11 ^b^	T159C, A238G, G255A, G279T, A330C, T336C (n = 3) ^a^
	*rrl*	11	De1954A (11) ^a^ Ins2004C (n = 1), C2078A (n = 1), Ins1977A (n = 1), Ins2779GC (n = 1), Ins2759G (n = 1)	11^b^	De1954A (11) ^a^, Ins2622A (n = 1), Ins2734G (n = 1), Ins1969C (n = 2), Ins1977A (n = 1), Ins2103C (n = 1), Ins2080C (n = 1)
Amikacin	*rrs*	10	Ins583T (n = 1)	11	Ins926T (n = 1), Ins812G (n = 1) Ins910G (n = 1), Ins891G (n = 1), C977T (n = 1)

Note

^a^ These mutations were also found in most susceptible strains (no association with MIC levels).

^b^ no sequence data available in 1 case. Serial isolates exhibiting both increase and decrease of MIC levels at different sampling times were included. The specified variants were relative to the previous isolate. Subspecies genetic markers were not included in the table. All *M*. *abscessus* from true infection cases (20 cases, 52 isolates) and colonization cases (6 cases, 16 isolates) were analyzed.

### Gene sequence analysis and drug resistant patterns among cases

For clarithromycin, *erm(41)* and *rrl* were analyzed with respect to subspecies (*massiliense* and non-*massiliense* subspecies). SNPs (C41A, A46G, G85T, C90T, G109A, A123G and A438C) and deletions (De61G, De62C and De156-429) of *erm(41)* were found in all *massiliense* subspecies, but G466A was not found in 4 isolates (**Table 5**). T159C and A330C were found in 27/29 isolates of non-*massiliense* subspecies. Twenty-two of 68 isolates (32.35%) had an inducible clarithromycin resistance phenotype and most of them (21/22, 95.45%) were from non-*massiliense* subspecies (**Table 5**). The *erm(41*) mutations associated with subspecies markers were significantly associated with MIC level of clarithromycin (**Table 6**). For *rrl*, 16 isolates exhibited acquired resistance to clarithromycin (**Table 5**); 7/16 isolates (46.67%) had *rrl* A2271G/C and 5/8 of these belonged to the *massiliense* subspecies (**Table 7**). Therefore, acquired resistance against clarithromycin mostly found in the *massiliense* subspecies and explained by *rrl* A2271G/C. *rrl* A2271G/C was also associated with clarithromycin MIC level (**Table 8**)

**Table 5:** Analysis between *erm(41)* gene sequence and clarithromycin susceptibility.

Patterns	n (%)	SNPs	
*Massiliense* subspecies (n = 35)			Indels
Acquired resistance (MIC≥8 μg/ml at day 3)	8 (22.86%)	- C41A, A46G, G85T, C90T, G109A, A123G, A438C, G466A (n = 8)	-De11A (n = 8) -De26A (n = 2) -De61G (n = 8) -De62C (n = 8) -De156-429 (n = 8)
Intermediate resistance (MIC = 4 μg/ml at day 5)	2 (5.71%)	- C41A, A46G, G85T,C90T, G109A, A123G, A438C, G466A (n = 1) - C41A, A46G, G85T, C90T, G109A, A123G, A438C (n = 1)	-De11A (n = 2) -De26A (n = 1) -De61G (n = 2) -De62C (n = 2) -De156-429 (n = 2)
Susceptible (MIC≤2 μg/ml at day 5)	24 ^a^ (68.57%)	- C41A, A46G, G85T, C90T, G109A, A123G, A438C (n = 1) - C41A, A46G, G85T, C90T, G109A, A123G, A438C, G466A (n = 22) - C41A, A46G, G85T, C90T, G109A, A123G, A438C, C54T, T55A (n = 1)	-De11A (n = 24) -De61G (n = 24) -De62C (n = 24) -De156-429 (n = 24) -De26A (n = 8)
Non-*massiliense* subspecies (n = 29)			
Inducible resistance (MIC≤2μg/ml at Day3 and MIC≥8 μg/ml at day 14)	21 (72.41%)	- T159C, A238G, G255A, A330C (n = 8) - T159C, A238G, G255A, G279T, A330C, T336C (n = 7) - T159C, A238G, G255A, G279T, A330C, T336C, A120G (n = 2) - T159C, A238G, G255A, G279T, A330C, T336C, C419T (n = 4)	-De11A (n = 19) -De26A (n = 3)
Acquired resistance (MIC≥8 μg/ml at day 3)	8 ^b^ (27.59%)	- A120G (n = 1) - G158A (n = 2) - T159C, A238G, G255A,A330C (n = 2) - T159C, A238G, G255A, G279T, A330C, T336C (n = 2) - T159C, A330C, C419T (n = 2)	-De11A(n = 7) -De26A(n = 2)
Any resistance	29 (100%)	- G158A (n = 2) - T159C, A238G, G255A, A330C (n = 8) - T159C, A238G, G255A, A330C, A120G (n = 2) - T159C, A238G, G255A, G279T, A330C, T336C (n = 11) - T159C, A238G, G255A, G279T, A330C, T336C, C419T (n = 4) - T159C, A330C, C419T (n = 2)	- De11A (n = 27) - De26A (n = 5)

Note: Sequence data were not available for 2/37 isolates belonging to the *massiliense* subspecies and 2/31 non-*massiliense* isolates

^a^ One isolate of the *massiliense* subspecies (defined based on *erm(41)* gene sequence) exhibited an inducible resistance pattern (susceptible at day 5 and became resistant at day 14).

^b^ There was 1 isolate that has MIC = 4 μg/ml at day 3 and then became resist at day 5 and day 14 was included as acquired resistant. All 64 isolates had T28 in the *erm(41)* gene.

**Table 6:** Comparison of SNPs of the *erm(41)* gene and MIC level for clarithromycin.

SNPs	Variants	n (%)	MIC (mean (SD))	P-values
C41A/A46G/G85T/C90T/G109A/A123G/ A438C, De61G De62 and De156-429 *	Wt	29 (45.31)	9.03 (6.09)	<0.0001
	Mut	35 (54.69)	4.24 (6.56)	
T159C /A330C	Wt	37 (57.81)	4.87 (6.92)	0.002
	Mut	27 (42.19)	8.51 (5.99)	
G279T/T336C	Wt	49 (76.56)	6.23 (7.14)	0.114
	Mut	15 (23.44)	7.00 (5.40)	

* *massiliense* subspecies marker. Wt: wild type (strains without specified mutations), Mut: mutant (strains with specified mutations).

**Table 7:** Association between *rrl* gene sequence and clarithromycin susceptibility in *M*. *abscessus* (n = 64).

Patterns	N (%)	SNPs	Indels
Resistant (MIC≥8 μg/ml at Day5)	27 (42.19)	- A2271G (n = 5) ^a^ - A2271C (n = 2) ^a^ - C2568T (n = 2) - C2695T (n = 1) - Ins2058G (n = 1) - C2078A (n = 1) - Ins2103C (n = 1) - Ins2080C (n = 1) - Ins1977A (n = 2) - Ins2004C (n = 3) - Ins2734G (n = 2) - Ins2759T (n = 1)	- De1954A (n = 27)
Intermediate (MIC = 4 μg/ml at Day5)	6 (9.40)	- Ins1969C (n = 1) - Ins2045C (n = 1) - C2078A (n = 1) - Ins2080C (n = 1) - Ins2103C (n = 2)	- De1954A (n = 6)
Susceptible (MIC≤2 μg/ml at Day5)	31 (48.44)	- Ins1969C (n = 1) - Ins1977A (n = 1) - Ins2004C (n = 1) - Ins2058G (n = 1) - C2078A (n = 1) - Ins2080C (n = 1) - Ins2080T (n = 1) - Ins2103C (n = 2) - Ins2622A (n = 1) - Ins2734G (n = 1) - Ins2759G (n = 1) - Ins2779GC (n = 1)	- De1954A (n = 31)

^a^ *rrl* SNPs conferring acquired resistance to clarithromycin were based on a previous study [6] and were found in 7/16 isolates with acquired resistance. Sequence data were not available for 4 of the 68 *M*. *abscessus* isolates. Twenty-seven clarithromycin-resistant isolates (based on results of drug susceptibility tests at day 5) comprised of 16 acquired resistant and 11 inducible resistant strains, 6 isolates with intermediate resistance against clarithromycin comprised of 2 that exhibited continuous intermediate resistance and 4 that exhibited inducible resistance (MIC≥8 μg/ml at day 14), 31 susceptible strains (based on day 5 test results) comprised of 24 susceptible strains and 7 that exhibited inducible resistant at day 14.

**Table 8:** Association between SNP of *rrl* gene and MIC level (μg/ml) for clarithromycin.

SNP	Variants	n (%)	MIC (mean (SD))	P-value
A2271G/C	A (wt)	57 (89.06)	5.23 (6.18)	0.001
	G/C	7 (10.94)	16.00 (0.00)	

For amikacin, no particular *rrs* variant was associated with resistance phenotype (**S2 Table**) or MIC level (**S3 Table**).

Raw data used in this study was available **S4 Table**.

## Discussion

*Mycobacterium abscessus* is an important pathogen in immunocompromised and immunocompetent patients causing pulmonary infection, soft tissue infections and disseminated infection [2]. *Mycobacterium abscessus* is one of the most antibiotic-resistant pathogens, rendering it difficult to treat [3]. Suggested antibiotics include clarithromycin, azithromycin, amikacin, cefoxitin, meropenem, imipenem, ciprofloxacin and trimethoprim/sulfamethoxazole, with clarithromycin being the key antibiotic [2]. It is likely that *M*. *abscessus* is intrinsically resistant to ethambutol, imipenem, isoniazid and rifampicin [26]. Ethambutol resistance is associated with alterations in *embB* [27]. Imipenem is highly unstable, thereby affecting/limiting *in-vitro* DST over several days, as required for *M*. *abscessus* [28]. Rifampicin resistance is due to the expression of a rifamycin ADP-ribosyltransferase (*arr*) [29]. Action of an efflux pump is a possible mechanism for intrinsic isoniazid resistance [3].

We have reported here the drug-resistance situation of *M*. *abscessus* infection in Northeast Thailand with implications for antibiotic selection for treatment. Different rates of clarithromycin resistance of *M*. *abscessus* have been reported; 75.34% in China [30], 35% in Venezuela [11], but resistance is rare in England [31]. We investigated the antibiotic resistance of *M*. *abscessus* in Northeast Thailand and found that, despite high levels of resistance to a broad range of antibiotics, clarithromycin remains the most effective antibiotic with >80% of isolates being susceptible. High or very high levels of susceptibility were noted to amikacin and tigecycline, which therefore are good candidates for treatment of *M*. *abscessus* infection in this region. The latter is not included in CLSI M24-A2 guidelines, yet half of the isolates had low MIC levels for tigecycline. Only 15% of isolates were susceptible to linezolid, rendering this antibiotic of limited value. However, tigecycline and linezolid were found to be the most effective combination for treatment tested in an animal model and humans [32]. We interpreted “high”, “moderate” and “low” resistance for non-CLSI standardized antibiotics based on the range of the MIC obtained from the studied strains for each drug. This approach could help to identify potential antibiotics for treatment and to monitor the drug resistance situation of *M*. *abscessus* infection.

*Mycobaterium abscessus* has been classified into three subspecies based on whole-genome sequencing analysis [4]. These subspecies differ in their susceptibility to clarithromycin [6, 33]. *Mycobacterium abscessus* subspecies *massiliense* does not show inducible resistance to clarithromycin [7]. Subspecies *abscessus* and *bolletii* seem to be able to resist clarithromycin through a mechanism depending on the *erm(41)* gene [6, 7, 34]. We analyzed the association between a panel of 15 antibiotics and *M*. *abscessus* subspecies in Northeast Thailand. We found that non-*massiliense* subspecies were significantly resistant to clarithromycin whereas the *massiliense* subspecies was significantly resistant to tigecycline. Resistance to tigecycline due to an alteration of tetracycline monooxygenase was recently reported [35]. Other mechanisms of tigecycline resistance might also exist. There is a risk that the *massiliense* subspecies might evolve greater resistance to tigecycline similar, in the way that the non-*massiliense* subspecies (*bolletii* and *abscessus* subspecies) have become highly resistant to clarithromycin. These findings could be helpful for managing antibiotic treatment of *M*. *abscessus* infection where subspecies information is available.

Previously, 2 deletions (nucleotides 64–65 and nucleotides 159–432) [7, 24, 25] and the mutation G466A in *erm(41)* [33] were suggested as markers for the *massiliense* subspecies. We found that 4 isolates of *massiliense* subspecies defined by these 2 deletions did not have *erm(41)* G466A. Furthermore, 1 out of the 4 isolates had an inducible resistance phenotype for clarithromycin, which should not be found in this subspecies. We also found a shift by 3 bp of the 2 deletions (nucleotides 64–65 and 159–432 became nucleotides 61–62 and 156–429, respectively). This shift implies a 3-bp deletion elsewhere in the upstream region of *M*. *abscessus* strains from our study.

Our study used serially sampled isolates from individual patients. Advantages of this approach include; (i) the genetic backbone is the same in each serially sampled strain, hence controlling for genetic differences between strains (ii) the change of MIC levels during the course of treatment can be used to study the response of the pathogen to the antibiotic. Furthermore, increasing or decreasing MIC levels can be used to study within-host evolution of a drug-resistant strain. Only a single previous study has investigated gene sequences of serially isolated strains of *M*. *abscessus*. However, this was a small study (10 isolates from 4 patients) and provided little information [22].

In the 2007 American Thoracic Society guidelines, macrolide drugs such as clarithromycin are recommended as key antibiotics for treatment of *M*. *abscessus* infection [2]. No previous study has investigate changes of MIC levels in clarithromycin and amikacin in association with sequences of drug-resistance genes. We investigated drug resistance in serially isolated strains of *M*. *abscessus* and sequenced *erm(41)* and *rrl* genes for clarithromycin and *rrs* gene for amikacin. These antibiotics were selected because they are the principal antibiotics used in our hospital for treatment of *M abscessus*, are recommended in the guidelines [2] and exhibit suitable variation of MIC levels. We analyzed genetic and MIC level changes during the course of persistent infection/colonization. In serially isolated strains, MIC levels for all antibiotics frequently changed between sampling times. The response of *M*. *abscessus* to antibiotics seems not to be stable through time, possibly in response to antibiotics previously used or other factors. Besides subspecies markers, no association was found between the mutations of *erm(41)*, *rrl*, and *rrs* genes and changes in MIC levels or the resistance phenotype for clarithromycin and amikacin.

Two mechanisms (acquired and inducible) of clarithromycin resistance have been reported. Acquired resistance to clarithromycin is associated with point mutations at A2271G [36] (or at 2058–2059 in the *Escherichia coli* numbering system [37, 38]) of the *rrl* gene. We found these two mutations only in 43.75% (7 strains) of all 16 isolates (5 of 8 *massiliense* and 2 of 8 non-*massiliense* subspecies isolates) with acquired clarithromycin resistance. It seems that acquired clarithromycin resistance in *M*. *abscessus* (of both subspecies) cannot be exclusively explained by *rrl* mutations. Concordantly, no A2271G variant of *rrl* was found in clarithromycin resistance strains from a previous study [22]. Therefore, A2271G *rrl* is not a robust resistant marker in our region and other associated mutations conferring resistance might exist.

T28C of *erm(41*) is associated with inducible resistance to clarithromycin [6, 7, 25] in non-*massiliense* subspecies [33]. Subspecies *massiliense* has 2 deletions leading to dysfunction of *erm(41)* and so the resistance mechanism is independent of T28 *erm(41)* and this subspecies is usually susceptible to clarithromycin [6, 7]. Our study concordantly found that all isolates of the *massiliense* subspecies had truncated *erm(41)* and 70% of them were susceptible to clarithromycin. For non-*massiliense* subspecies (subspecies without *erm(41)* deletion markers), only strains containing T28 *erm(41)* exhibit inducible clarithromycin resistance [33]. All of our non-*massiliense* subspecies isolates had T28C in *erm(41*) and around 75% of these had inducible clarithromycin resistance. So inducible clarithromycin resistance in our non-*massiliense* subspecies can be explained by T28C in *erm(41*). However, one isolate of the *massiliense* subspecies (identity based on 2 deletions in *erm(41)*) showed an inducible resistance phenotype (MIC at day 3 = 0.12 μg/ml, at day 14 = 16 μg/ml) in our study. A recent study reported that some *M*. *abcessus* strains with a functional *erm(41)* gene did not exhibit a clarithromycin inducible resistance phenotype: hence the presence of a functional *erm(41)* gene should not be used as a marker for inducible clarithromycin resistance [39]. However, our study found a rare strain with a non-functional *erm(41)* gene but exhibiting the inducible resistance phenotype. It is therefore unclear whether the inducible clarithromycin resistance phenotype is specific to non-*massiliense* subspecies or not and inducible resistant mechanisms other than the *erm(41)* gene might exist. Whole-genome sequencing analysis of these phenotypically diverse strains could unveil such complexity. Interestingly, we found that inducible and acquired resistance to clarithromycin in *M*. *abscessus* were interchangeable during the course of infection or colonization. This new finding was only possible because we applied DST to serial *M*. *abscessus* isolates from patients.

Amikacin is one of the antibiotics recommended for treatment of NTM infection and *M*. *abscessus* infection. It is a bactericidal antibiotic based on inhibition of protein synthesis though alteration of the 16S rRNA. Mutations of the *16S rRNA* gene (*rrs*) at T1406A, A1408G, C1409T and G1491T are reportedly responsible for high resistance levels to amikacin [8, 14, 15]. We did not find any of these mutations associated with amikacin resistance in *rrs*, implying that other, undetected mutations may be responsible for conferring resistance in our region. A recent study reported an additional mechanism of amikacin resistance in *M*. *abscessus*. This was associated with mutations of *aac(2ʹ)* and *eis2* [40]. High-throughput genomic analysis should provide an insight into the genetic mechanisms involved.

The strains used in our study were genotyped using multi-locus sequence-typing (MLST) (unpublished data, work under review). Based on 24 patients (62 isolates), only two clonal clusters (2 isolates from 2 patients and 8 isolates from 5 patients) were found. We recruited an additional two patients for the current study. The presence of such clusters may bias comparisons of phenotypic and genotypic DTS. We classified *M*. *abscessus* into *massiliense* and non-*massiliense* subspecies only based on sequence deletions in *erm(41)*. However, the MLST data (unpublished data) showed that the *bolletii* subspecies was not among our studied strains. Drug-susceptibility tests based on broth microdilution antibacterial assays were performed according to RAPMYCOI Sensititre’s protocol. Although the CLSI guidelines do not specify the control strain or expected DST results for *M*. *abscessus*, our DST results were similar to those in a previous study of *M*. *abscessus* ATCC19977 [41].

In conclusion, *M*. *abscessus* was highly resistant to almost all antibiotics, but highly susceptible to amikacin and moderately susceptible to clarithromycin and tigecycline. Non-*massiliense* subspecies were associated with clarithromycin resistance whereas the *massiliense* subspecies tended towards tigecycline resistance. Around 75% of non-*massiliense* subspecies had inducible clarithromycin resistance explicable by T28C of *erm(41*) but inducible clarithromycin resistance might not be specific to non-*massiliense* subspecies only. The recognized mutation of the *rrl* gene (A2271GC) conferred acquired resistance to clarithromycin in only half of the strains. No mutation of the *rrs* gene associated with amikacin resistance in *M*. *abscessus* was found during our study.

## Supporting information

S1 Table Changes in MIC levels of serially isolated strains of *M*. *abscessus* (n = 26 cases). Note: In 4 cases (2 cases of infection/reinfection and 2 cases of colonization/recolonization) different strains were isolated during the study (based on MLST data (Kham-ngam I. et al, unpublished data). These 4 cases have been excluded from analysis. S = Susceptible, I = Intermediate, R = Resistant, SXT = Trimethoprim/sulfamethoxazole, AMC = Amoxicillin/clavulanic acid.
(DOCX)

S2 Table Association between *rrs* gene sequences and amikacin susceptibility. Note: S = Susceptible, I = Intermediate, R = Resistant.
(DOCX)

S3 Table Association between SNPs of the *rrs* gene and MIC level of amikacin. Note: These 2 highly variable SNPs were selected for SNPs-MIC association analysis.
(DOCX)

S4 Table Raw data used in this study. (XLSX)
