# Supplementary material for: Analysis of drug-susceptibility patterns and gene sequences associated with clarithromycin and amikacin resistance in serial Mycobacterium abscessus isolates from clinical specimens from Northeast Thailand
Source: PLoS One. 2018 Nov 29;13(11):e0208053. doi: 10.1371/journal.pone.0208053 (PMC6264522; doi:10.1371/journal.pone.0208053)
Supplement: S1 Table — Note: In 4 cases (2 cases of infection/reinfection and 2 cases of colonization/recolonization) different strains were isolated during the study (based on MLST data (Kham-ngam I. et al, unpublished data). These 4 cases have been excluded from analysis. S = Susceptible, I = Intermediate, R = Resistant, SXT = Trimethoprim/sulfamethoxazole, AMC = Amoxicillin/clavulanic acid. (DOCX) [file pone.0208053.s001.docx]

**S1 Table. Changes in MIC levels of serially isolated strains of *M. abscessus* (n=26 cases).**

| Antibiotics | Changes of MIC  (cases) | | | Changes of susceptibility  (cases) | | |
| --- | --- | --- | --- | --- | --- | --- |
|  | Increased MIC | Decrease MIC | Both changes of MIC | S to R conversion | R to S conversion | Both changes of R and S pattern |
| Amikacin | 7 | 7 | 2 | 3 | 2 | 1 |
| Cefoxitin | 3 | 4 | 1 | 0 | 0 | 0 |
| Ciprofloxacin | 1 | 2 | 0 | 0 | 0 | 0 |
| Clarithromycin | 5 | 6 | 5 | 3 | 3 | 1 |
| Doxycycline | 2 | 3 | 0 | 0 | 0 | 0 |
| Imipenem | 1 | 4 | 0 | 0 | 0 | 0 |
| Linezolid | 3 | 7 | 3 | 0 | 0 | 0 |
| Moxifloxacin | 3 | 2 | 0 | 0 | 0 | 0 |
| Tobramycin | 3 | 5 | 2 | 0 | 0 | 0 |
| SXT | 1 | 2 | 0 | 0 | 0 | 0 |
| Minocycline | 2 | 3 | 1 | 0 | 0 | 0 |
| Tigecycline | 8 | 11 | 3 | 0 | 0 | 0 |
| Cefepime | 1 | 2 | 0 | 0 | 0 | 0 |
| AMC | 1 | 2 | 0 | 0 | 0 | 0 |
| Ceftriaxone | 1 | 2 | 0 | 0 | 0 | 0 |

Note: In 4 cases (2 cases of infection/reinfection and 2 cases of colonization/recolonization) different strains were isolated during the study (based on MLST data (Kham-ngam I. et al, unpublished data). These 4 cases have been excluded from analysis. S=Susceptible, I=Intermediate, R=Resistant, SXT=Trimethoprim/sulfamethoxazole, AMC=Amoxicillin/clavulanic acid.
