# Supplementary material for: Analysis of drug-susceptibility patterns and gene sequences associated with clarithromycin and amikacin resistance in serial Mycobacterium abscessus isolates from clinical specimens from Northeast Thailand
Source: PLoS One. 2018 Nov 29;13(11):e0208053. doi: 10.1371/journal.pone.0208053 (PMC6264522; doi:10.1371/journal.pone.0208053)
Supplement: S2 Table — Note: S = Susceptible, I = Intermediate, R = Resistant. (DOCX) [file pone.0208053.s002.docx]

**S2 Table. Association between *rrs* gene sequences and amikacin susceptibility**

| Patterns | n (%) | SNPs | Indel |
| --- | --- | --- | --- |
| R | 3 (4.69) | C977T (n=1) | - |
| I | 7 (10.94) | C977T (n=1)  Ins583T (n=1) | - |
| S | 54 (84.38) | - A52T, A53C, C56A, G57T (n=1) - Ins798G (n=1) - Ins807T (n=1) - C809T (n=1) - T811C (n=1) - Ins812G (n=2)  - Ins812T (n=1)  - Ins851G (n=1) - Ins891G (n=4) - Ins894G(n=1) - Ins910G (n=1) - Ins919G (n=2) - G921T (n=1) - Ins926T (n=7) - Ins929G (n=1) - A976G (n=11) - C977T (n=19) | De54G (n=1) |

Note: S=Susceptible, I=Intermediate, R=Resistant.
