# Supplementary material for: Analysis of drug-susceptibility patterns and gene sequences associated with clarithromycin and amikacin resistance in serial Mycobacterium abscessus isolates from clinical specimens from Northeast Thailand
Source: PLoS One. 2018 Nov 29;13(11):e0208053. doi: 10.1371/journal.pone.0208053 (PMC6264522; doi:10.1371/journal.pone.0208053)
Supplement: S3 Table — Note: These 2 highly variable SNPs were selected for SNPs-MIC association analysis. (DOCX) [file pone.0208053.s003.docx]

**S3 Table. Association between SNPs of the *rrs* gene and MIC level of amikacin**

| SNPs | Bases | n (%) | MIC (mean (SD)) | P-values |
| --- | --- | --- | --- | --- |
| A976G | A | 53 (82.81%) | 15.25 (14.76) | 0.086 |
|  | G | 11 (17.19%) | 8.00 (3.10) |  |
| C977T | C | 43 (67.19%) | 14.33 (14.24) | 0.590 |
|  | T | 21 (32.81%) | 13.33 (12.98) |  |
| Note: These 2 highly variable SNPs were selected for SNPs-MIC association analysis | | | | |
